# Patient and Therapist In-Session Cortisol as Predictor of Post-Session Patient Reported Affect

**DOI:** 10.3390/brainsci11111483

**Published:** 2021-11-10

**Authors:** Eyal Levi, Susanne Fischer, Hadar Fisher, Roee Admon, Sigal Zilcha-Mano

**Affiliations:** 1 School of Psychological Sciences, University of Haifa, Mount Carmel, Haifa 31905, Israel; eyallevi122@gmail.com (E.L.); hadar.fisher@gmail.com (H.F.); radmon@psy.haifa.ac.il (R.A.); 2 Institute of Psychology, Clinical Psychology and Psychotherapy, University of Zurich, 8050 Zürich, Switzerland; s.fischer@psychologie.uzh.ch; 3 The Integrated Brain and Behavior Research Center (IBBRC), University of Haifa, Haifa 31905, Israel

**Keywords:** affect, cortisol, psychotherapy, social support, stress, therapist

## Abstract

The importance of the role of affect in psychotherapy for major depressive disorder (MDD) is well established, but the common use of self-reported measures may limit our understanding of its underlying mechanisms. A promising predictor of patient affect is the stress hormone cortisol. To date, no studies have studied in-session changes in cortisol in psychotherapy for MDD. We investigated whether an increase in patient cortisol over the course of a session correlated with higher negative and lower positive affect. Given previous findings on healthy individuals on the contagious nature of stress, an additional aim was to examine whether these relationships are moderated by therapist cortisol. To this end, 40 dyads (including 6 therapists) provided saliva samples before and after four pre-specified sessions (616 samples). After each session, the patients provided retrospective reports of in-session affect. We found no association between patient cortisol and affect. However, increases in patient cortisol predicted negative affect when the therapists exhibited decreases in cortisol, and increases in patient cortisol predicted positive affect when the therapists showed increases. Our study provides initial evidence for the importance of the social context in the cortisol–affect relationship in MDD.

## 1. Introduction

According to the World Health Organization, major depressive disorder (MDD) is a leading cause of disability worldwide and a main contributor to the overall global burden of disease [1,2]. Thus, a wide research array is devoted to study psychotherapy for MDD, a standard first-line treatment for depressive disorders [3,4,5]. Studies have nominated affect as a fundamental agent of change in psychotherapy, either as a predictor for therapeutic processes [6] or treatment outcome [7]. For example, one study reported that the smaller the ratio of momentary positive to momentary negative affect at the beginning of psychotherapy, the less likely patients were to make progress in the initial five sessions of psychotherapy [8]. Furthermore, a meta-analysis attested to a significant decline in negative and a significant rise in positive affect over the course of psychotherapy for MDD [9]. These findings highlight the importance of learning more about the underpinnings of affective states in psychotherapy.

However, many studies in psychotherapy settings face limitations as they are based on self-report measures or an external evaluator. For example, self-report assessment methods may be biased to varying degrees, as they rely on the patient’s motivation or insight to identify and report on their affect [10]. While the clinical relevance of affect has been well established in psychotherapy for MDD, the sole reliance on self-report measures could limit the understanding of underlying mechanisms. Thus, it may be of value to integrate objective methods to inspect affect processes occurring in psychotherapy, such as sampling the stress hormone cortisol. It seems noteworthy that, to date, the occasions in which biological markers have found their way into psychotherapy research are still rare. In MDD, the main focus has been on the treatment level, where the “hypercortisolism” that is often present in patients [11] has been found to predict unfavorable treatment outcomes [12,13] and to “normalize” over the course of treatment [14,15]. Thus, inspecting affect processes in psychotherapy for MDD through cortisol sampling could both shed more light on the mechanism of change at work and contribute to the growing field of biological markers research in psychotherapy.

In recent years, the stress hormone cortisol, the end product of the hypothalamic-pituitary–adrenal axis, has emerged as a potential indicator of affect processes. Cortisol is reliably secreted in situations that pose a threat to the social self, with peak concentrations observed after approximately 20 min [16]. Cortisol subsequently crosses the blood–brain barrier and, by binding to mineralocorticoid and glucocorticoid receptors expressed in the limbic system, is capable of modulating the emotional experience [17]. In line with these findings, a meta-analysis in healthy adults found that increases in cortisol in everyday life were associated with small but significant increases in negative affect and small but significant decreases in positive affects [18]. These links were generally confirmed in an ambulatory assessment study, which followed individuals with MDD for 30 days [19]. However, notably, the strength of the cortisol–affect relationship was far from being uniform across individuals. These findings could be interpreted from a contextual perspective, meaning that the very context where the cortisol–affect relationship takes place may serve as a condition for their occurrence.

Indeed, a study in healthy adolescents has found that a previously observed cortisol-negative affect association vanished under conditions of high social support [20], one of the most potent stress-buffering factors [21]. Therefore, it is conceivable that the extent to which the social environment (e.g., romantic partners, friends, or other types of significant others) is responsive to situations causing a cortisol increase in the depressed individual may be a crucial modulator of their affective state. Interestingly, experimental research suggests that this responsiveness may manifest on the biological level, such that the significant other not only experiences “empathic stress”, but also exhibits higher cortisol levels [22]. Further in line with these notions, psychosocial stress has repeatedly been shown to enhance emotional empathy [23,24,25,26], but see [27]. When taken together, it is thus conceivable for the depressed individual’s and significant other’s cortisol levels to co-predict the depressed individual’s affect. More specifically, it seems possible that an increase in cortisol is less likely to map onto negative affect when this physiological change is mirrored by the significant other. Psychotherapy may serve as an ideal setting to study to what extent the cortisol–affect relationship may be moderated by social factors, as it is inherently social and more controlled than everyday life settings, in which the majority of past research was conducted. Moreover, research measuring patient affect during psychotherapy is scarce [28] and no studies have, to date, examined to what extent patient and therapist cortisol may interact with patient in-session affect. This is all the more important, since individuals are rarely aware of the degree to which they exhibit a bodily stress response [29].

**Figure 1:**
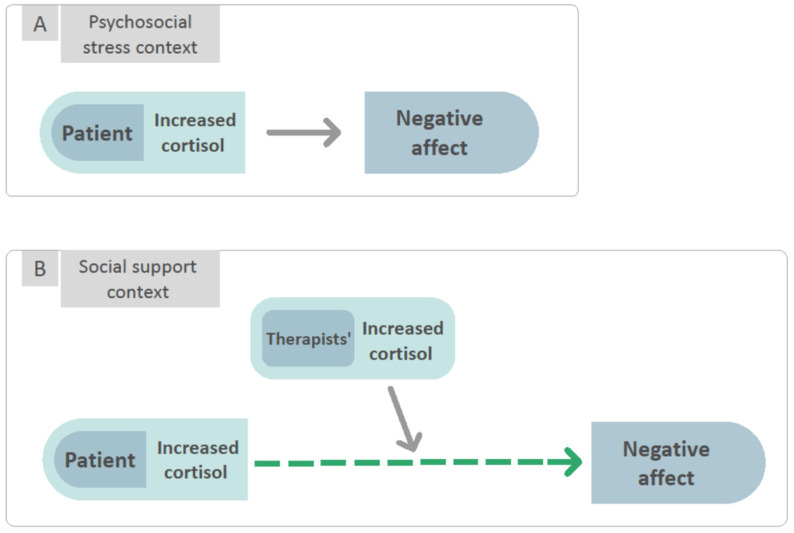
In line with our hypotheses, when in the context of psychosocial stress, there exists an association between increased patient cortisol levels and negative affect. Alternatively, when in the context of social support, the association between increased cortisol levels and negative affect is diminished when the therapist exhibits increased cortisol levels as well. Psychosocial stress context (**A**) is defined as an interpersonal situation that results in increased cortisol levels. Social support context (**B**) is defined as the empathic stress response of the other in an interpersonal situation.

The aim of the present study was to investigate these potential relationships for the first time, in a sample of patients with MDD undergoing state-of-the-art psychodynamic psychotherapy. To this end, the patients and therapists were asked to provide saliva samples for the determination of cortisol before and after four pre-specified sessions, resulting in 616 observations in total. Moreover, after each session, the patients provided retrospective reports of in-session affect by means of a validated questionnaire. Based on a prior study in MDD (see above), we expected that the greater a patient’s increase in cortisol over the course of the session, (1) the higher their negative affect scores and (2) the lower their positive affect scores. Furthermore, referring to the above literature on empathic stress, enhanced stress-induced empathy, and the stress-buffering effects of social support, we assumed that (3) the higher the therapist’s cortisol level, the weaker the patient’s cortisol-negative affect relationship (Figure 1). In addition, we were interested in exploring whether similar moderating effects were present regarding the cortisol-positive affect relationship.

## 2. Materials and Methods

### 2.1. Participants and Treatments

Forty patients with MDD were recruited through advertisements offering free treatment for depression. Inclusion criteria were (a) meeting MDD diagnostic criteria according to structured clinical interviews for the fifth edition of the Diagnostic and Statistical Manual of Mental Disorders [30], scores above 14 on the 17-item Hamilton Rating Scale for Depression [31] at two evaluations 1 week apart, and current MDD as measured by the Mini-International Neuropsychiatric Interview [32]; (b) any medication taken by patients had to have been dosage stable for at least three months before entering the study, and they had to be willing to maintain a stable dosage for the duration of the treatment; (c) age between 18 and 60 years; (d) Hebrew language fluency; and (e) provision of written informed consent. Exclusion criteria were (a) current risk of suicide or self-harm (HRSD suicide item >2); (b) current substance abuse disorder; (c) current or past diagnosis of schizophrenia or psychosis, bipolar disorder, or severe eating disorder requiring medical monitoring; (d) history of organic mental or bodily disease; and (e) participating in psychotherapy in the last three months. Patients were enrolled in 16-session manualized psychotherapy for MDD, as part of a randomized control trial [33,34]. The study was approved by the relevant Institutional Review Board, and informed consent was obtained from all participants involved in the study. For each patient, therapy sessions occurred at a fixed time and day of the week. A total of 608 saliva samples, collected from patients and their therapists, were used in the analyses. Samples were taken from patients at intake, and from both patients and therapists at treatment sessions 4, 8, 12, and 16. At a fixed time of the day per participant, two samples were collected in each treatment session, 30 min before therapy and immediately after (pre-session and post-session). The mean patient age was 30.78 (SD = 10.42), and 21 patients were female. At intake, the mean patient BMI score was 24.65 (SD = 6.02), and 5 patients were using anti-depressant medication along with treatment. Participants were asked to refrain from eating, drinking (other than water), smoking, and intimate contact with others (e.g., hugging) for at least 30 min before the saliva sample procedure.

### 2.2. Therapists

Six therapists, all with formal training in psychodynamic treatment and at least five years of experience, participated in the current study. Therapists completed 20 h workshop training in short-term psychodynamic techniques used in the RCT. Therapists received individual and group supervision both while training and during the active phase of the RCT. Five of the therapists were women. Overall, therapists had an average age of 42.33 (SD = 4.41) and an average of 14.41 years of clinical experience (SD = 5.42).

### 2.3. Procedure and Measures

#### 2.3.1. Salivary Cortisol

Participants were asked to place cotton swabs in their mouths, provided in Sarstedt Salivette^®^ (Nümbrecht, Germany) containers, for 2 min. Participants were asked to report in an online questionnaire about possible factors which may affect cortisol levels at the time the sample was taken (e.g., medication use, alcohol or drug consumption, menstrual cycle phase, contraceptives use). Saliva samples were stored at −20 °C until analysis. Samples were delivered in dry ice, under temperature control maintenance, to the daacro GmbH & Co. KG lab, University of Trier, Trier, Germany, where they were thawed, vortexed, and analyzed in duplicates to assure the reliability of results. Sample Intra- and inter-assay coefficients of variance (CV) were calculated. Intra-assays CV of less than 10% and inter-assay CVs of less than 15% are generally acceptable [35]. For both patients and therapists, Cortisol change throughout treatment was calculated in delta scores (post-session minus pre-sessions scores), for each session individually.

#### 2.3.2. Patient Positive and Negative Affect Experiences

Patient positive and negative affect experiences were measured at post-session, using the Positive and Negative Affect Scale [36], a self-report questionnaire consisting of 20 items. Ten items assess subjective experience of positive affect (e.g., interested, enthusiastic, active), and ten items assess subjective experience of negative affect (e.g., irritable, upset, scared). For the analysis, we used PANAS post-session scores of sessions 4, 8, 12, and 16, concurrent with the cortisol measurements during the RCT. At post-session, patients received the following instructions: “In the following questionnaire, you will find a number of words, each describing a different feeling or emotion. Read each word and rate to what extent you have experienced this feeling during the current session.” Items were rated on a scale from 1 (very low or not at all) to 5 (very much). Individual scores were calculated for positive affect and for negative affect, for each session (Sessions 4, 8, 12, 16) throughout treatment. The internal reliability range for positive affect was 0.87–0.94, and the internal reliability range for negative affect was 0.82–0.89.

### 2.4. Data Analysis Strategy

The data were hierarchically nested on three levels: assessments nested within patients nested within therapists. Thus, we employed a multi-level models (MLM) approach using SAS PROC MIXED [37]. To determine the proportion of variance in negative affect (NA) and positive affect (PA) accounted for by the random effects of the therapist we used intraclass correlation coefficients (ICCs). Therapist effect was null in all analyses (estimate = 0.00, *p* = 0.99), and therefore, two-level models with sessions nested within patients were used.

To examine NA and PA behavior over time, we evaluated the linear and linear in log of time trend models as fixed or random effects for each. We started with a model with only a fixed intercept and no random effects and added sequentially a random intercept, fixed effect of week, and random effect of week in therapy. Next, we examined the models with fixed and random linear effects of log of week. We used the log-likelihood test and the Akaike information criterion (AIC) to determine whether the inclusion of each term improved the model fit. The model found to have the best fit based on the AIC for NA and PA was the one with only a fixed effect of log of time and random intercept. This model was used in all subsequent analyses. To disentangle the within-person from the between-person effects, the predictor variables (patients’ and therapists’ cortisol change score) in the models were centered on each patient’s mean. Standardized effect sizes for each variable in the model were calculated by standardizing the raw variables and re-running the models and may thus be regarded as an approximation of standardized betas [38]. Descriptive statistics for all the variables are shown in Table 1.

## 3. Results

### 3.1. Patients’ Cortisol as a Predictor of NA and PA

To test whether changes in patients’ cortisol predict NA and PA reported by the patients following each session, the following Level-1 equation was estimated:

PA/NA*_sp_* = β_0_ + β_1_ × Pcortisol*_sp_* + β_2_ × Logtime*_sp_* + e*_sp_* (1)

where patients negative or positive affect reported at the end of session *s* by patient *p* was predicted by (a) patient’s intercept (β_0_); (b) the difference score of patients’ cortisol (Pcortisol = patients’ cortisol, calculated as post-session minus pre-session cortisol) at session *s* (β_1_); (c) the effect of time in treatment (β_2_); and a Level-1 residual error (e*_sp_*). A first-order auto-regressive structure was imposed on the level-1 residual covariance matrix. At Level 2, the intercept was considered to be random (i.e., effects were allowed to vary between patients). The results of these models are presented in Table 2. In contrast to our first hypothesis, patients’ cortisol change did not predict NA nor PA.

### 3.2. To Examine the Interaction between Patients’ and Therapists’ Cortisol in Predicting NA and PA Reported by the Patients Following Each Session

To Test whether the Interaction between Patients’ and Therapists’ Cortisol Predict NA and PA Reported by the Patients following Each Session, the following Level-1 Equation Was Estimated

PA/NA*_sp_* = β_0_ + β_1_*Pcortisol*_sp_* + β_2_*Tcortisol*_sp_* + β_3p_*Pcortisol_sp_*Pcortisol*_sp_* + β_4_*Logtime + e*_sp_*

where patients NA or PA reported at the end of session *s* by patient *p* was predicted by (a) patient’s intercept (β_0_), (b) the difference score of patients’ cortisol (post-session minus pre-session cortisol) at session *s* (β_1_), (c) the difference score of therapists’ cortisol at session *s* (β_2_), (d) the interaction between patients’ and therapists’ cortisol difference scores at session *s* (β_3_), (e) the effect of time in treatment (β_4_), and a Level-1 residual error (e*_sp_*). A first-order auto-regressive structure was imposed on the Level-1 residual covariance matrix. At Level 2, the intercept was considered to be random. The results of this model are presented in Table 3.

**Figure 2:**
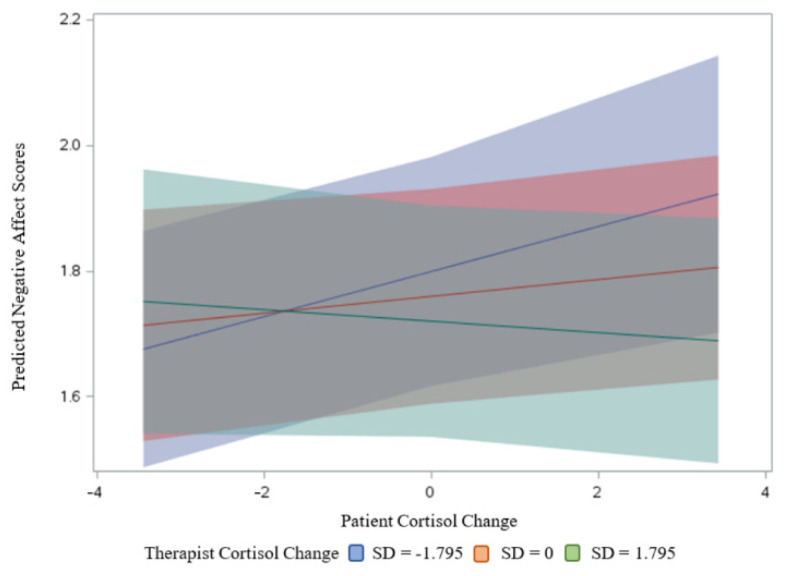
Interaction of patient and therapist cortisol change predicting negative affect. Note. The relationship between patients’ cortisol change and NA was positive and significant when their therapists showed smaller increases (or greater decreases) in cortisol (represented in blue), but this association was diminished when their therapists showed greater increases in cortisol (represented in green).

For NA, there was a significant interaction effect between patients’ and therapists’ cortisol change in predicting NA (Figure 2). Given the significant interaction effect, we also tested the simple slopes. Simple slope analyses demonstrated that the relationship between patients’ cortisol change and NA was positive and significant (estimate = 0.035, *SE* = 0.014, *t* = 2.58, *p* = 0.01) when their therapists showed smaller increases (or greater decreases) in cortisol (e.g., 1 SD below the mean), but this association was diminished (estimate = −0.009, *SE* = 0.013, *t* = −0.72, *p* = 0.47) when their therapists showed greater increases in cortisol (e.g., 1 SD above the mean) (Figure 2) (while controlling for the existence of PTSD diagnosis, gender of patients, therapists, and same dyad gender, we found the same pattern of results).

**Figure 3:**
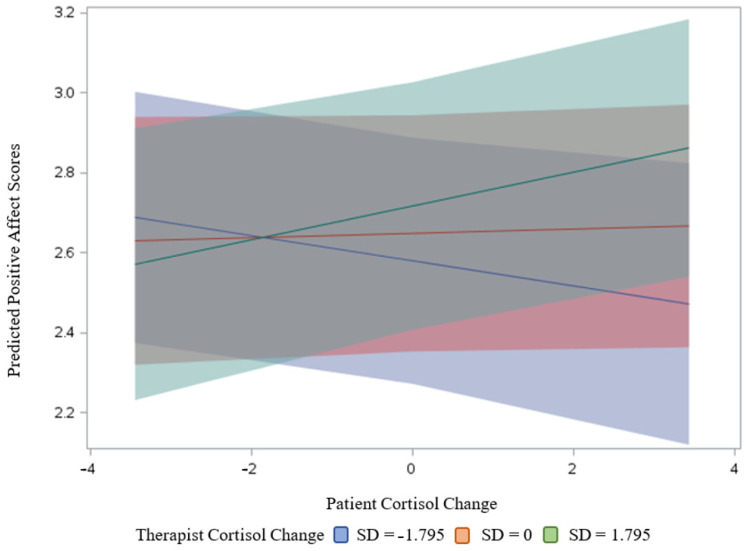
Interaction of patient and therapist cortisol change predicting positive affect. *Note.* The relationship between patients’ cortisol change and PA was positive and significant when their therapists showed greater increases in cortisol (represented in green), but not significant when their therapists showed smaller increases (or greater decreases) in cortisol (represented in blue).

For PA, as in the case of NA, there was a significant interaction effect between patients’ and therapists’ cortisol change in predicting PA (Figure 3). The results of simple slope tests demonstrated that the relationship between patients’ cortisol change and PA was positive and significant (estimate = 0.037, *SE* = 0.017, *t* = 2.58, *p* = 0.03) when their therapists showed greater increases in cortisol (e.g., 1 SD above the mean), but not significant (estimate = −0.036, *SE* = 0.019, *t* = −1.83, *p* = 0.07) when their therapists showed smaller increases (or greater decreases) in cortisol (e.g., 1 SD below the mean) (Figure 3) (while controlling for the existence of PTSD diagnosis, gender of patients, therapists and same dyad gender, we found the same pattern of results).

## 4. Discussion

The aim of the present study was to investigate whether patient and therapist cortisol may serve as conjoint predictors of patient affect as experienced in psychotherapeutic sessions. Two of the four hypotheses we had were supported. Although we did not receive support for a general effect of cortisol change during sessions on patient post-session negative affect (Hypotheses 1–2; Figure 1A), we found a significant moderating effect of therapist cortisol change during therapy sessions (Figure 1B). Specifically, supporting our third hypothesis, the relationship between patients’ cortisol change and NA was positive and significant when their therapists showed smaller increases (or greater decreases) in cortisol, but this association was diminished when their therapists showed greater increases in cortisol. Supporting our fourth hypothesis, the relationship between patients’ cortisol change and PA was positive and significant when their therapists showed greater increases in cortisol but not significant when their therapists showed smaller increases (or greater decreases) in cortisol.

Concerning negative affect, our findings only partly align with our hypotheses. In contrast to Hypothesis A, in which we had expected that the greater a patient’s increase in cortisol, the higher their negative affect, such a main effect was not supported. This finding contradicts prior research in healthy adults see [18] for a meta-analysis and a previous study in MDD [19], which, overall, found small correlations between cortisol and negative affect. The most likely explanation for this discrepancy is the study setting. Whereas prior research was conducted in everyday life contexts, our setting was inherently social. Therefore, it likely provided more “opportunities” for the cortisol-negative affect association to be modulated by potent external factors. In line with this notion and with Hypothesis C, which stated that the cortisol-negative affect relationship in patients would be diminished when the therapists had high levels of cortisol, our results indicated that cortisol only predicted negative affect when the therapist exhibited comparably low cortisol levels. This finding dovetails with the literature on empathic stress [22], on enhanced stress-induced empathy [23,24,25,26], but see [27], and on social support as a stress-buffering factor [20], which suggests increased cortisol to indicate empathy and social support. Future studies can further explore this explanation by testing the interaction between patients’ cortisol change and patient-therapist alliance in predicting patient affect.

Concerning positive affect, the present findings contradict Hypothesis B, in which we stated that the greater a patient’s increase in cortisol over the course of the session the lower their positive affect, as no main effects of cortisol on positive affect emerged. By contrast, exploratory analyses revealed a positive link between cortisol and positive affect, which was conditional on the therapist exhibiting relatively high cortisol levels. As outlined in the previous paragraph, this result attests to the necessity of considering contextual information when attempting to unravel the intricate interlinks between stress and affect.

When taken together, our findings imply that cortisol can map onto either negative or positive affect in depressed individuals, depending on the social environment they find themselves in. More specifically, based on these findings, we can speculate that elevated cortisol in patients throughout psychotherapy is coupled with their negative affect, which may manifest as distress, nervousness, or hostility in sessions in which the therapist exhibits low cortisol, and thus might be relatively relaxed. In contrast, elevated cortisol in patients throughout psychotherapy is coupled with their positive affect, which may manifest as strength, elatedness, and activity when the therapist is exhibiting high cortisol. One interpretation of this finding is that the more resonant the therapist is to the patient’s stress, the more pleasant the patient affect during the session. Alternatively, the role of affect in psychotherapy is unique in that activating and experiencing a wide range of emotions, both positive and negative, is a key ingredient of therapeutic success [39]. This means that, contrary to other settings, certain levels of positive affect are not necessarily desirable nor particularly beneficial for the therapeutic process, while the experience of negative affect, although unpleasant, may foster beneficial therapeutic processes [40]. Further studies combining observational coding with saliva sampling and affect ratings are now warranted to attribute further meaning to the herein found associations. In addition, even more fine-grained sampling schedules will be necessary to establish a temporal order between patient and therapist cortisol and affect. Together with symptomatic outcome measures, such approaches may ultimately shed light on how the biological attuning with their therapist is involved in the patient’s affective development over the course of psychotherapeutic treatment.

Considering their relative scarcity, the study adds to a burgeoning field of research that attempts to exploit biological markers as an additional avenue towards our understanding of in-session therapeutic dynamics [41,42,43,44]. The general importance of a holistic approach towards illness has repeatedly been highlighted, and many are the arguments supporting a biopsychosocial approach to mental disorders [45]. The fact that cortisol can now be determined non-invasively and in an increasing number of tissues [29,46,47] is promising and could pave the way for a more multidimensional depiction of patient (and therapist) states in clinical practice in the future.

Our study presents a number of strengths. First and foremost, it adds to the scant literature investigating how cortisol relates to affect, a key perpetuating factor of MDD. It extends this literature not only by means of setting (i.e., psychotherapy), but also by means of design (i.e., dyadic). Second, ours was a homogenous sample of patients with moderate to severe depression, and we were able to eliminate or control a number of important confounders of cortisol (e.g., comorbidity with major mental disorders, such as substance abuse, schizophrenia, or eating disorders, time of day). This attests to the reliability of our data. Third, our statistical approach allowed us to account for the nested structure of the data (i.e., the therapeutic dyads), as well as to disentangle the trait-like component of cortisol from its state-like analog. However, a number of limitations are equally important to mention. First, the size of the present sample was relatively small, hence limiting its representativeness. Second, we relied on retrospective reports of in-session affect, which may be subject to memory bias. Third, in the absence of audio/video recordings analyses, it remains unknown what events drove the observed increases/decreases in cortisol in the patients and therapists. Fourth, and related to this, this was a correlational study, and further research will be necessary to unravel the temporal dynamics between patient and therapist cortisol and affect. Finally, the present study was conducted as part of a larger trial on the efficacy of specific forms of psychodynamic psychotherapy. This means that our findings do not necessarily generalize to other types of social contexts.

## 5. Conclusions

In sum, the present study provides initial evidence for cortisol to map onto either negative or positive affect in depressed individuals, depending on the cortisol changes of the therapist, implying the importance of the social context. Specifically, for NA, the relationship between patients’ cortisol change and NA was positive and significant when their therapists showed smaller increases (or greater decreases) in cortisol, yet this relationship diminished when the therapists showed greater increases in cortisol. Regarding PA, the relationship between patients’ cortisol change and PA was positive and significant when their therapists showed greater increases in cortisol, but not when their therapists showed smaller increases (or greater decreases) in cortisol. Further research is warranted to explore to what degree the biologically attuning of significant others may be a co-determinant of affective states in depression.

## Figures and Tables

**Table 1:** Means, standard deviations, intercorrelations and distribution of variables.

Variable	1	2	3	4	M (SD)	Skew	Kurtosis
Negative affect	**-**				1.54 (0.58)	1.46	1.95
Positive affect	−0.29 **	-			2.91 (0.93)	0.15	−0.46
Patients’ cortisol	0.10	0.09	-		−2.46 (4.95)	−0.82	3.26
Therapists’ cortisol	−0.11	−0.04	0.17 *	-	−0.80 (2.16)	−0.45	6.21

* *p* < 0.05. ** *p* < 0.01.

**Table 2:** Parameter estimates for patients’ cortisol change predicting negative affect (Model 1) and positive affect (Model 2).

	Model 1 Outcome: NA			Model 2 Outcome: PA		
Fixed Effects	b (SE)	*p*	Effect Size	b (SE)	*p*	Effect Size
Intercept	2.12 (0.15)	<0.001		2.65 (0.15)	<0.0001	
Patients’ cortisol	0.01 (0.01)	0.10	0.09	0.00 (0.01)	0.60	0.02
Log time	−0.26 (0.06)	<0.001	−0.24	0.26 (0.09)	0.004	0.14

*Note.* NA = Negative affect; PA = Positive affect.

**Table 3:** Parameter estimates for the interaction between patients’ and therapists’ cortisol change predicting negative affect (Model 1) and positive affect (Model 2).

	Model 1 Outcome: NA			Model 2 Outcome: PA		
Fixed Effects	b (SE)	*p*	Effect Size	b (SE)	*p*	Effect Size
Intercept	1.76 (0.09)	<0.001		2.65 (0.15)	<0.0001	
Patients’ cortisol	0.01 (0.01)	0.14	0.09	0.00 (0.01)	0.28	0.00
Therapists’ cortisol	−0.02 (0.02)	0.25	−0.07	0.04 (0.03)	0.18	0.07
Patients’*therapists’ cortisol	−0.01 (0.00)	0.02	−0.13	0.02 (0.01)	0.026	0.14
Log time	0.03 (0.01)	<0.001	−0.27	0.03 (0.01)	0.001	0.15

*Note.* NA = Negative affect; PA = Positive affect.

## Data Availability

Regarding the data Availability, when this research was carried out, the informed consent form for the participants stated that we would keep the data strictly confidential. Therefore, if uploading data, we must seek consent from our participants and the consent of the ethics committee. Therefore, the data are not currently available.
